# Dengue Virus-Susceptible Animal Models: Research Progress, Core Bottlenecks, and Future Perspectives

**DOI:** 10.3390/vaccines14040319

**Published:** 2026-04-03

**Authors:** Wensheng Zhang, Yue Zhao, Teng Meng, Yuling Tang, Yifei Zhang, Lu Zhang, Shoulong Deng, Yan Li, Yiming Yuan, Yefeng Qiu

**Affiliations:** 1 Academy of Military Medical Sciences, Academy of Military Sciences, Beijing 100850, China; 2 National Science Center for Model Animals, China Agricultural University, Beijing 100193, China

**Keywords:** dengue virus, susceptible animal models, pathogenic mechanism, ADE

## Abstract

Dengue fever (DF) is an acute mosquito-borne infectious disease caused by dengue virus (DENV), primarily transmitted by Aedes aegypti and Aedes albopictus. Nearly 4 billion people worldwide are at risk of infection, and the 2024 epidemic reached an unprecedented scale. Severe cases can lead to hemorrhage, shock, and even death, prompting the WHO to classify it as a potential pandemic pathogen. Current prevention and control measures face prominent bottlenecks, including limited applicable populations for vaccines, lack of specific antiviral drugs, and increasing insecticide resistance in mosquito vectors. Notably, susceptible animal models serve as core tools for elucidating the pathogenic mechanisms of dengue virus, screening antiviral drugs, and evaluating vaccine protective efficacy, holding irreplaceable significance. This review systematically summarizes the characteristics, application scenarios, and research progress of mainstream and potential susceptible animal models, including non-human primates, mice, pigs, tree shrews, and bats. It covers model systems with different immune statuses, genetically modified types, and species-specific traits. Among these, mouse models are the most widely used due to their high flexibility and controllable cost, while non-human primate models have become key carriers for preclinical vaccine evaluation by virtue of their high homology with human immune responses. However, current models generally suffer from core bottlenecks, such as incomplete simulation of core severe phenotypes, insufficient restoration of immune mechanisms, unclear viral receptor mechanisms, and lack of unified standards for inoculation doses and evaluation indicators. These limitations make it difficult to accurately replicate key severe disease mechanisms, including antibody-dependent enhancement (ADE) and cytokine storms. Future model development should focus on core requirements—including intact immunity, broad-spectrum susceptibility, and accurate simulation of clinical pathological features—prioritize solving the simulation challenges of ADE and cytokine storms, and establish standardized experimental systems and evaluation criteria. By comprehensively summarizing the advantages and limitations of the existing models, this review provides a systematic reference for the optimization and upgrading of dengue virus-susceptible animal models. It also holds important guiding significance for promoting the in-depth development of basic dengue research, innovation in prevention and control technologies, and clinical transformation and application.

## 1. Dengue Fever

Dengue fever (DF) is an acute mosquito-borne viral disease caused by dengue virus (DENV), primarily transmitted by Aedes aegypti and Aedes albopictus [1]. It poses a serious threat of mosquito-borne infectious diseases in tropical and subtropical regions worldwide. Clinical phenotypes of DF vary significantly: most infected individuals show no obvious symptoms or only mild symptoms, which are mainly high fever, headache, myalgia, arthralgia, and rash, with a self-limiting course overall. A small proportion (0.5–5%) of cases can progress to severe forms such as dengue hemorrhagic fever (DHF) and dengue shock syndrome (DSS) [2,3], accompanied by life-threatening manifestations including plasma leakage, bleeding tendency, and multiple organ damage.

### 1.1. Epidemic Status of Dengue Fever

**Figure 1:**
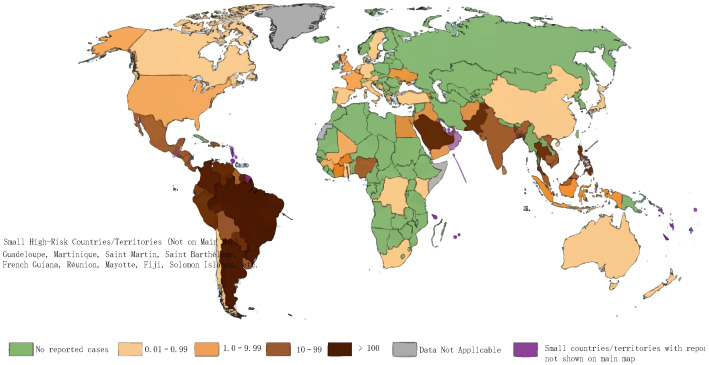
2023 global reported dengue incidence rate map.

Dengue fever is an acute mosquito-borne infectious disease transmitted by Aedes aegypti and Aedes albopictus through bites. Patients and asymptomatic infected individuals are the main sources of infection. Currently, the global epidemic presents a severe pattern of heavy burden and continuously expanding spread [1,4]. According to the data released by the World Health Organization (WHO) in August 2025, approximately 390 million people are infected with DENV annually worldwide, of which 96 million develop clinical symptoms, 500,000 require hospitalization due to severe cases, and 20,000 die. Nearly 4 billion people live in epidemic risk areas, accounting for nearly half of the global population. The 2024 global epidemic reached an unprecedented high, with more than 80 countries/regions reporting over 10 million cases and more than 6000 deaths, representing a two-fold increase in incidence compared with the same period in 2023 [5,6], as shown in Figure 1.

The reported dengue incidence rate per 100,000 population in countries/regions worldwide in 2023 is classified into five risk categories based on severity: no reported cases (green), low incidence rate (0.01–0.99 cases per 100,000 population, light beige), low-to-moderate incidence rate (1.0–9.99 cases per 100,000 population, light orange), moderate-to-high incidence rate (10–99 cases per 100,000 population, dark brown), and high incidence rate (>100 cases per 100,000 population, dark sepia). Gray indicates data not available, and purple marks small countries/regions with reported cases that are not displayed on the main map (compiled based on data from the World Health Organization (WHO) and the European Centre for Disease Prevention and Control (ECDC)) [7].

In terms of geographical distribution, traditional endemic areas of dengue fever are concentrated in the eastern Mediterranean, Southeast Asia, Africa, the Western Pacific and South America. The Southeast Asia and Western Pacific regions have maintained a high epidemic level for a long time. Countries such as Vietnam, the Philippines, and Nepal have experienced frequent surges in cases in recent years, among which the American region has become the hardest-hit area. In 2024 alone, Brazil reported 6.5 million suspected and confirmed cases with over 6300 deaths. Large-scale outbreaks also occurred in countries like Argentina and Honduras [7]. Moreover, the epidemic scope has continuously broken through the boundaries of tropical and subtropical regions, gradually spreading to temperate regions: many European countries have witnessed the colonization and continuous spread of Aedes albopictus, the transmission vector. The number of local infection cases in the EU has increased by approximately 78.1% compared with the cumulative number from 2010 to 2021, and imported cases have also shown a significant upward trend [8,9]. In China, the epidemic is concentrated in southern port cities; local outbreaks occur annually in Guangdong, Hainan, Taiwan, and other regions [10]. In 2024, Shenzhen and Guangzhou experienced local outbreaks with a week-on-week increase of over 260% in cases, and cities such as Foshan launched emergency mosquito control operations in 2025 due to rising mosquito density.

In 2024, the WHO reincluded DENV in the list of potential next pandemic pathogens, highlighting its persistent threat to the global public health system [7]. Current epidemic response still faces the shortcoming of uneven monitoring. In low-income areas, insufficient testing resources lead to underreporting of cases, and the high proportion of asymptomatic infected individuals also results in a serious underestimation of the actual infection burden [11]. At the same time, some countries have launched emergency responses due to uncontrolled epidemics. In September 2024, Bohol Province in central Philippines declared a state of calamity due to a surge in cases. Global prevention and control are currently in a dilemma of “predominantly passive response and insufficient active blocking”, and there is an urgent need to upgrade prevention and control strategies to curb the spread [12].

### 1.2. Characteristics of Dengue Virus

Dengue virus belongs to the Flaviviridae family and Orthoflavivirus genus. It is a group of single-stranded positive-sense RNA viruses with conserved morphological structures, and its biological characteristics are highly homologous to those of other Orthoflavivirus members such as Japanese encephalitis virus and Zika virus [13]. Viral particles are typically spherical with a diameter of approximately 50 nm, surrounded by a lipid envelope. Two key structural proteins are embedded on the envelope surface, namely, the E protein (envelope glycoprotein) and the M protein (membrane protein). The interior consists of an icosahedrally symmetric nucleocapsid, which is formed by the tight binding of the C protein (capsid protein) and genomic RNA. The overall structure is stable, and it has morphological markers unique to the Orthoflavivirus genus [14].

The viral genome is approximately 10.7 kb in length, with an m7GpppAmp-type cap structure at the 5’ end and no polyadenylate tail at the 3’ end. Both ends of the genome are untranslated regions (UTRs), and there is only one open reading frame (ORF) in the middle, encoding a full-length polyprotein. This polyprotein is co-cleaved by host cell proteases and viral NS3 protease to produce three structural proteins (C, PrM, and E) and seven non-structural proteins (NS1, NS2A, NS2B, NS3, NS4A, NS4B, and NS5). Among them, the PrM protein is the precursor form of M protein, which is cleaved into the functional M protein during maturation. As an RNA virus, dengue virus lacks a proofreading mechanism during replication and has a high mutation rate, which is also the core cause of genotypic diversity within serotypes [13,15].

Currently, four dengue virus serotypes (DENV-1~DENV-4) have been clearly identified, among which DENV-1~DENV-4 are the classic serotypes. The genomic sequence homology among each serotype is approximately 65–70%, with significant differences in antigenicity. Infection with one serotype can only produce lifelong specific immunity to that serotype, and it only provides short-term partial protection to other serotypes. In addition, the first new dengue virus serotype in 50 years was officially announced by the research team led by Nikos Vasilakis from the University of Texas Medical Branch at the International Dengue Conference in Bangkok. This serotype circulates mainly among non-human primates (macaques) in the forests of Sarawak, Malaysia, belonging to the sylvatic transmission cycle, which is distinct from the human-to-human transmission cycle of DENV-1 to DENV-4. The viral sample was isolated from the blood specimen of a 37-year-old local male farmer. To date, this serotype has only been linked to a mild outbreak in Sarawak, Malaysia, in 2007, without establishing a sustained human-to-human transmission chain. It is speculated that the virus has circulated in the jungles of Malaysia and Indonesia for tens of thousands of years, and the human infection detected represented a sporadic cross-host spillover event [16].

### 1.3. Pathogenic Mechanism of Dengue Virus

**Figure 2:**
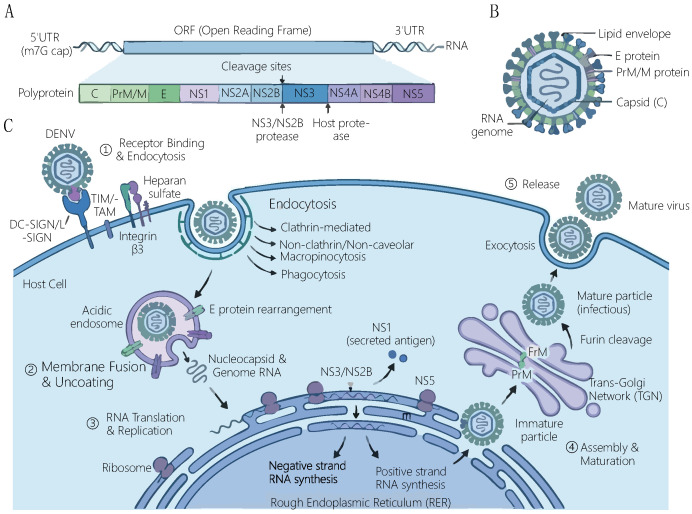
Schematic representation of the genome structure and replication cycle of dengue virus. (**A**) The genome and polyprotein structure of dengue virus. (**B**) The virion structure of dengue virus. (**C**) The complete life cycle of dengue virus infecting host cells.

As illustrated in Figure 2, dengue virus (DENV) has a characteristic flavivirus genome structure, virion morphology, and a complete life cycle of infecting host cells, which encompasses the entire process from viral entry, replication, assembly to release.

The pathogenic process of dengue virus is a complex network of interactions between the virus and the host, which mainly follows the molecular pathway of “receptor binding–cell invasion–immune escape–tissue damage”, and it is regulated by factors such as viral serotype and host immune status. The first step of viral invasion depends on the specific binding to receptors on the surface of host cells. Known main receptors include dendritic cell-specific intercellular adhesion molecule-3-grabbing non-integrin (DC-SIGN) [17], Fcγ receptors (FcγR) [18], and integrin β3 [19,20]. Among them, DC-SIGN mediates the initial infection of antigen-presenting cells such as dendritic cells and macrophages by the virus, while FcγR plays a key role in ADE. During secondary infection, cross-reactive antibodies produced by the host bind to the virus to form immune complexes, which mediate viral entry into macrophages through FcγR and lead to massive viral replication in the cells.

After entering the cell, the virus forms endosomes through clathrin-mediated endocytosis. Acidification of endosomes triggers conformational changes in the E protein, mediating the fusion of the viral envelope with the endosomal membrane and releasing genomic RNA into the cytoplasm [21]. Subsequently, viral RNA is translated and replicated on the surface of the rough endoplasmic reticulum. The NS5 protein, as an RNA-dependent RNA polymerase, catalyzes viral genome synthesis. At the same time, NS3 protease participates in polyprotein cleavage, and non-structural proteins such as NS1, NS2A, and NS4B collaboratively construct replication complexes and promote viral replication by regulating host cell signaling pathways. During replication, the virus evades host immune surveillance through multiple mechanisms: NS5 protein can inhibit STAT2 phosphorylation and promote its degradation, blocking the type I interferon (IFN) signaling pathway [22]; NS4A binds to the MAVS protein, interfering with RIG-I/MAVS-mediated antiviral responses; subgenomic RNA (sfRNA) produced by the viral 3′UTR inhibits the host RNAi pathway and regulates the expression of host metabolic genes.

Recent studies have found that the DB structure of dengue virus can promote immune escape by regulating the expression of host immune genes. Viruses lacking the DB structure significantly upregulate the expression of defensin C (DefC) in the mosquito midgut, and DefC can directly inhibit viral replication and block its breakthrough of the midgut barrier. This mechanism reveals a new dimension of interaction between viral RNA structure and host immunity. In addition, sphingosine secreted by Enterobacter hormaechei strain B17, an intestinal symbiotic bacterium, can block dengue virus invasion during the viral fusion stage. Its molecular mechanism is to bind to the viral E protein or change membrane curvature, inhibiting the fusion process between the virus and the host cell membrane, which provides a new perspective for understanding the regulation of host flora on viral pathogenesis [23].

Primary DENV-2 infection invades host cells through the hTim4 receptor, inducing the overexpression of the Syk protein [24,25]. On the one hand, it drives Th2-polarized cytokine storm (significant upregulation of core factors such as IL-6 and IL-10) to damage vascular integrity. On the other hand, it regulates coagulation-related genes (such as Col1a1 and Col3a1) to cause coagulation–fibrinolysis imbalance, manifesting as disseminated intravascular coagulation (DIC) characteristics such as thrombocytopenia and prolonged activated partial thromboplastin time/prothrombin time (APTT/PT) and ultimately leading to severe phenotypes such as multiple organ hemorrhage, vascular leakage, and microcirculatory failure. This pathway has been verified by clinical samples to be highly consistent with the pathological mechanism of human DHF/DSS. It is clarified that primary infection can independently induce severe cases through Syk-mediated immune coagulation cross-disorder, revising the understanding from a single driver to multi-mechanism synergy [24].

ADE is the most distinctive immunopathological phenomenon of dengue fever and the biggest obstacle to vaccine development. The occurrence of human ADE has strict temporal sequence and conditions: sub-neutralizing heterotypic cross-reactive antibodies are produced 3–12 months after the initial infection. At this time, secondary infection with heterotypic DENV occurs, and virus–antibody complexes enter monocytes/macrophages through Fc receptors, leading to a 10–100-fold increase in viral replication and causing severe DHF/DSS. The core pathological mechanism of severe dengue fever is closely related to the cytokine release syndrome (cytokine storm) induced by the ADE effect.

### 1.4. Clinical Phenotypes of Dengue Fever

The clinical phenotypes of dengue fever are significantly heterogeneous, mainly divided into classic dengue fever (DF), severe dengue (SD), and phenotypes involving specific organs. Among them, classic phenotypes account for the highest proportion (approximately 80–90%). Although the incidence of severe phenotypes is only 5–10%, the mortality rate is significantly increased. Phenotypes involving special organ complications are often overlooked due to their insidious clinical manifestations, and they are important factors affecting prognosis [26,27].

The incubation period of classic dengue fever is 3–14 days, and the typical course is self-limiting, divided into three stages: febrile phase, critical phase, and recovery phase. The febrile phase is characterized by sudden high fever (body temperature ≥ 39 °C) as the initial symptom, accompanied by systemic symptoms such as severe headache, retro-orbital pain, myalgia, arthralgia (breakbone fever), fatigue, nausea, and vomiting. Some patients develop maculopapular or morbilliform rashes. The critical phase mostly occurs on days 3–8 of the course. Symptoms gradually relieve in mild cases, while high-risk groups for severe disease (infants, the elderly, pregnant women, patients with secondary heterotypic serotype infection, and those with underlying diseases such as hypertension and diabetes) may show warning signs, including persistent vomiting, severe abdominal pain, mucosal bleeding, drowsiness, irritability, and hepatomegaly (>2 cm). In the recovery phase, body temperature returns to normal, rashes subside, and some patients experience skin itching and desquamation. Laboratory examinations show decreased white blood cell count and platelet count [28,29,30,31].

According to the WHO 2009 [32] classification criteria, the core characteristics of severe dengue (SD) are severe plasma leakage, severe bleeding, or severe organ damage. Its pathological basis is increased systemic capillary permeability caused by vascular endothelial injury, which is closely related to the excessive immune response induced byADE. Among them, in addition to fever, patients with dengue hemorrhagic fever (DHF) also present with thrombocytopenia (platelet count ≤ 100 × 10^9^/L) and hemoconcentration (hematocrit increase >20%). Clinically, it can present with superficial bleeding such as skin ecchymoses, gingival bleeding, and epistaxis. Severe cases may have massive gastrointestinal bleeding or intracranial hemorrhage. Dengue shock syndrome (DSS) is a critical stage of DHF, manifested by shock symptoms such as rapid pulse, narrowed pulse pressure, hypotension, cold extremities, and confusion. Without timely fluid resuscitation treatment, the mortality rate can reach 10–20%. SD patients are often complicated with multiple organ function damage. The incidence of acute kidney injury (AKI) is 2.74% in ordinary DF cases and as high as 35.12% in SD cases, and the higher the AKI stage, the higher the mortality risk (the mortality risk of stage 3 AKI is 10.77 times higher than that of patients without AKI) [29,31].

Dengue fever may also present with a variety of rare phenotypes involving special organ impairment, which are often superimposed on severe conditions, significantly increasing the difficulty of diagnosis and treatment, as well as the risk of death, and thus require enhanced clinical monitoring. Renal complications are mainly manifested as acute kidney injury, caused jointly by direct viral damage and immune complex deposition; severe cases may require renal replacement therapy, and some patients may develop residual chronic renal insufficiency during the recovery period [33,34]. Although neurological complications, including dengue encephalitis, Guillain–Barré syndrome, and other types, have a relatively low incidence of 0.5% to 6.2%, they are life-threatening, with a mortality rate of approximately 5% to 10%; survivors are prone to residual sequelae such as cognitive and motor dysfunctions [35,36]. In addition, phenotypes such as acute liver injury, myocarditis, and hemolytic anemia (more prevalent in patients with G6PD deficiency) may also be observed.

### 1.5. Vaccines, Therapeutic Drugs, and Preventive Measures

At present, the prevention and control system of dengue fever is based on a prevention-first approach, supplemented by treatments, but the existing measures still have significant limitations, and it is difficult to contain the spread of the epidemic. In terms of vaccines, three dengue fever vaccines have entered the application stage worldwide. CYD-TDV (Dengvaxia™, Sanofi Pasteur, Lyon, France) is the first globally licensed live-attenuated chimeric tetravalent dengue vaccine. Clinical studies have demonstrated that the vaccine is associated with a significantly elevated risk of severe dengue and increased hospitalization rates in seronegative children who have not been previously infected with dengue virus, leading to strict restrictions on its use and its current discontinuation of production. TAK-003 (Takeda Pharmaceutical, Tokyo, Japan) is a tetravalent live-attenuated vaccine, prequalified by WHO in 2024, suitable for children aged 6–16 years, and approved in 41 countries, but there is a large supply gap. Butantan DV (Instituto Butantan, São Paulo, Brazil) is the world’s first single-dose vaccine, approved in 2025 for people aged 12–59 years, with a protective efficacy of 91.6% against severe disease; its large-scale supply is expected in 2026 [37,38,39]. However, all existing vaccines are live-attenuated vaccines, which are not suitable for immunocompromised individuals and pregnant women. They may also have ADE risks in people who have not been infected with dengue fever, limiting their scope of application.

In terms of treatment, there is still no specific antiviral drug in clinical practice, and symptomatic and supportive treatment is the core. For fever management, physical cooling or acetaminophen is preferred. Non-steroidal anti-inflammatory drugs such as aspirin are strictly prohibited to avoid increasing the risk of bleeding. Fluid replacement therapy is the key to the treatment of severe cases. Mild cases receive oral rehydration, while severe cases require intravenous volume expansion to maintain water and electrolyte balance and prevent shock. For patients with bleeding tendency, coagulation function should be monitored, and platelets or coagulation factors should be supplemented if necessary [40]. Experimental antiviral drugs such as mosnodenvir (clinical trials show preventive potential) and nucleoside analog NITD008 are still in the research stage, lacking large-scale clinical data verification, and they have not been approved for application.

Preventive measures focus on mosquito vector control. Traditional measures include environmental management (removal of water-holding containers), chemical mosquito control (insecticide spraying), and physical protection (mosquito nets, repellents). However, long-term use of chemical insecticides has induced insecticide resistance in mosquitoes, limiting the effectiveness of prevention and control [41]. New biological prevention and control technologies, such as releasing Aedes mosquitoes carrying Wolbachia and engineered intestinal symbiont AS1-TK, have been piloted in many countries, and they have shown potential for transmission blocking, but they still need official certification by the WHO to promote global popularization.

In general, the limited applicable population of existing vaccines, the lack of specific antiviral drugs, and the challenges of insecticide resistance and technology promotion in mosquito vector control have left dengue fever prevention and control in a passive situation. As core tools for elucidating pathogenic mechanisms, screening antiviral drugs, and evaluating vaccine protective efficacy, susceptible animal models have problems such as insufficient accuracy in simulating pathological characteristics of human infection and incomplete simulation of ADE effect. These problems have become key bottlenecks restricting the breakthrough of dengue fever prevention and control technologies. Therefore, constructing more ideal dengue virus-susceptible animal models has important scientific research and clinical transformation value.

## 2. Dengue Virus-Susceptible Animal Models

### 2.1. Non-Human Primate (NHP) Models

Non-human primates are the only known vertebrates naturally infected with DENV besides humans. Their immune responses are highly similar to those of humans, serving as a key bridge between preclinical research and clinical trials.

Among them, rhesus monkeys, cynomolgus monkeys, and marmosets are the main research objects. Studies have shown that after subcutaneous injection of 10^5^ PFU dengue virus into rhesus monkeys (simulating the inoculation dose of mosquito bites), the virus can replicate in lymph-rich tissues such as lymphoid tissues, but the viral load is significantly lower than that in humans [42,43]. Rhesus monkeys treated with cyclophosphamide (an immunosuppressant) can achieve long-term dengue virus infection of monocytes and prolong the viral replication cycle [44]. However, in most cases, there are no obvious clinical symptoms. Only some rhesus monkeys occasionally show thrombocytopenia, accompanied by mild laboratory index changes such as lymphadenopathy, lymphocytosis, and leukopenia [45]. Fumihiro et al. first evaluated the feasibility of bonnet macaques (Macaca radiata) as a non-human primate model for dengue virus infection. They screened out dengue virus type 4 (DENV-4) strain 09-48 through in vitro PBMCs. After intravenous inoculation of 1 × 10^6^ PFU into three bonnet macaques, high-titer viremia (peak 2.2–4.0 × 10^6^ copies/mL), NS1 antigen positivity, human-like antibody responses (IgM appearing at 3–5 dpi, IgG appearing at 11–14 dpi), and transient leukopenia and thrombocytopenia were observed. No obvious clinical symptoms were found, confirming that bonnet macaques can serve as a reliable model for dengue virus research, especially suitable for the evaluation of vaccines and antiviral drugs [46].

Azami systematically compared the dengue virus infection characteristics of three NHPs: cynomolgus monkeys, common marmosets, and tamarins. The results showed that common marmosets were superior in viral load (peak 6.3 ± 1.0 log10 genome copies/mL), antibody response (IgM appearance time 4.1 ± 1.5 days), and clinical symptom simulation, making them a more reliable model for vaccine evaluation [47]. Benjamin included the primary dengue virus (DENV) infection data of 11 non-human primate (NHP) species from 54 experiments (including three unpublished studies). He clarified that DENV-2 had the longest viremia duration in rhesus monkeys (median 5.13 days), and DENV-4 had the shortest viremia duration (median 3.13 days). There was a significant negative correlation between viral inoculation dose and viremia duration (for each 1 log10 PFU increase, the duration shortened by 0.44 days). There were no significant differences in the onset time and duration of viremia among different NHP species except patas monkeys. The duration of viremia detected through RT-PCR and IFA was significantly longer than that detected through the plaque formation assay. These results provide key parameters for dengue vaccine development and forest-type dengue transmission dynamics modeling [48]. Nattawat successfully induced typical dengue hemorrhagic symptoms (petechiae and subcutaneous hematomas appearing at 3–5 days) in six rhesus monkeys through intravenous injection of 1 × 10^7^ PFU per animal of dengue virus type 2 (strain 16681), establishing the first non-human primate model that can reproduce the core characteristics of human dengue hemorrhagic fever (DHF). Key pathological changes such as peak viral load of 10^6^ copies/mL (3–4 days), mild thrombocytopenia, neutropenia, significant elevation of D-dimer, and biphasic aggregation of platelet-monocytes/neutrophils (1–3 days and 7 days after infection) were observed in the model, confirming that monocytes phagocytosing virus antigen-containing platelets may be involved in the pathogenesis [49]. Virus strains isolated from non-human primates are genetically different from those infecting humans, indicating that these transmission chains have long been differentiated [50].

Non-human primates are currently widely used in the research of candidate dengue virus vaccines because they develop acute viremia and produce strong neutralizing antibody responses after viral challenge, providing irreplaceable empirical support for vaccine development [47,51,52,53]. Among them, rhesus monkeys, as the core model for preclinical evaluation of dengue fever vaccines, are widely used in the verification of immunogenicity, protective effect, and safety [51,54]. Cynomolgus monkeys are used for vaccine immune response analysis and challenge experiments, adapting to the evaluation of various vaccine types [55,56]. Daniel Thoresen et al. developed a tetravalent dengue virus-like particle (DENVLP) vaccine and conducted experiments in two non-human primate models: cynomolgus monkeys and common marmosets. The results showed that the vaccine could induce high-titer neutralizing antibodies against all four dengue virus serotypes, with a protective period of up to 1 year after immunization. No ADE effect was detected in vitro. The immunization with the vaccine could significantly reduce the viral replication level in marmosets after the infection. The passive transfer of IgG from immunized cynomolgus monkeys could protect AG129 mice from lethal dengue virus challenge, confirming that its protective effect depends on humoral immunity. This vaccine provides strong support for the clinical evaluation of a new generation of dengue fever vaccines [57]. Therefore, non-human primates are widely used for vaccine testing.

### 2.2. Mouse Models

#### 2.2.1. Immunocompetent Mouse Models

Neonatal Mouse Models (C57BL/6, BALB/c strains): The establishment of this model originated in the 1990s. Since adult immunocompetent mice can quickly clear DENV and cannot establish infection, researchers found that neonatal mice (≤7 days old) are susceptible to DENV due to immature immune systems (insufficient IFN-α/β secretion and imperfect T cell function). In 2021, Byrne et al. further optimized the model [58], confirming that 3-day-old neonatal mice are of the optimal modeling age and establishing standardized operating procedures (such as inverse IP injection technology). Its core advantage is retaining immunocompetent characteristics, which can simulate age-related susceptibility characteristics of infant dengue fever. It is safe to operate and has high repeatability, allowing stable observation of pathological manifestations such as elevated liver enzymes and viral dissemination in organs. The limitation is that there is no obvious thrombocytopenia after the infection, and the mice survive stably until day 8, but it cannot simulate the bleeding and shock symptoms of severe dengue fever. The core significance of this model is to provide a naturally immunocompetent model close to clinical practice for studying the pathogenesis of infant dengue fever, age-related differences in immune responses, and early viral organ damage, filling the gap in models for infant dengue fever research.

Adult Immunocompetent Mouse Adapted Strain Models: The construction of this model can be traced back to a 2012 study by Denise Gonçalves et al. [59]. In this study, immunocompetent C57BL/6 mice developed dengue-related manifestations after intraperitoneal inoculation with DENV-1 Mochizuki strain (a neuroadapted strain), including viremia, elevated liver enzymes, thrombocytopenia, splenic hemorrhage, and increased levels of cytokines such as IFN-γ and TNF-α. Its advantages lie in the absence of immunosuppressive treatment, simple operation, and low cost, making it suitable for the preliminary screening of antiviral drugs. The limitations of this model are relatively mild pathological manifestations (e.g., dependence on specific serotypes and inoculation routes), and there are difficulties in simulating core severe dengue symptoms such as vascular leakage. Its significance is to provide immune interference-free model support for evaluating antiviral drugs, analyzing DENV replication kinetics and studying host innate immune regulation, thereby facilitating the development of basic antiviral strategies.

Wild-Type Neonatal Mouse Intranasal Infection Non-Invasive Dengue Encephalitis Models (C57BL/6, BALB/c strains): Based on the defect that traditional neonatal mouse intraperitoneal/intracranial infection models cannot simulate the peripheral-to-central infection process in humans, 3-day-old C57BL/6 and BALB/c neonatal mice were used [60]. DENV non-adapted strains were inoculated intranasally to simulate the viral invasion of the central nervous system (CNS) through the olfactory pathway. Its core advantage is that no invasive inoculation is needed, and it can reproduce the natural process of dengue encephalitis patients from peripheral infection to central involvement. It can stably observe pathologies such as neuronal loss and CNS inflammation (upregulation of IL-1β, IL-6, and CCL family chemokines), and the virus has significant brain tissue tropism, with the peak viral load in the brain much higher than that in other organs. The limitation is that it is only applicable to neonatal mice (immature immune system), it cannot simulate the pathological changes in central infection in adult individuals, and no obvious bleeding or shock symptoms occur, focusing only on nervous system damage. The significance of this model is to fill the gap in non-invasive dengue encephalitis models, provide a more clinically relevant tool for studying DENV neurotropic mechanisms and central nervous system damage pathways (such as Fas/FasL-mediated neuronal apoptosis), and facilitate the evaluation of central protective effects of dengue encephalitis-related drugs and vaccines [61,62].

#### 2.2.2. Immunodeficient Mouse Models

SCID Mice (C.B-17 Background, T/B Cell Deficiency): The model originated in 1983 when a research team at the Fox Chase Cancer Center in the United States discovered it from C.B-17 inbred mice [63]. It is caused by a recessive SCID gene mutation on chromosome 16, resulting in a significant reduction in T lymphocytes and B lymphocytes in the thymus, spleen, and lymph nodes, and defects in cellular and humoral immune functions, but macrophages and NK cells function normally. In the 1990s, the U.S. Naval Medical Research Center first used it for dengue fever research. After reconstructing the immune system through intravenous injection of human peripheral blood lymphocytes, DENV-1 was used for challenge. Its advantage is that it can partially reconstruct the human immune environment, providing an early tool for studying the interaction between human-specific immunity and DENV, and it can achieve immunodeficient phenotypes without gene editing. The limitation is that the onset time after infection is scattered (24–53 days), the incidence rate is only 13–28%, the sensitivity to DENV is low, and effective infection can only be established by relying on immune cell transplantation [64]. It is susceptible to opportunistic pathogen infection during feeding. The significance is that, as the first dengue fever-related mouse model that can reconstruct human immunity, it laid the foundation for the development of improved models such as NOD/SCID, promoted the start of research on human-specific immune responses to dengue fever, and provided the practical basis for the exploration of early humanized immune models.

NOD/SCID Mice (NOD/Lt × SCID Hybrid): The model was developed by a team at the Southwest Foundation for Biomedical Research in the United States by crossing non-obese diabetic mice (NOD/Lt) with SCID mice to obtain a double-mutant mouse strain [65]. It not only inherits the T/B cell deficiency of SCID mice but also has lower NK cell activity, resulting in a more complete immunodeficiency. An infection model can be established by transplanting human CD34+ umbilical cord blood hematopoietic stem cells through the tail vein and then intraperitoneally injecting DENV-2 [66]. The advantage is that the immunodeficiency is more comprehensive, and the low NK cell activity reduces the rejection of transplanted human cells. After the infection, it can show clinical symptoms similar to human dengue fever (DF) such as fever, rash, and thrombocytopenia [67], with viremia lasting 2–6 days. Viral RNA can be detected in the spleen, liver, and skin, and the symptoms are close to the clinical natural infection. The limitation is that it still relies on human hematopoietic stem cell transplantation to simulate human immune responses, the feeding cost is high, and the requirements for environmental cleanliness are strict. It cannot be used for long-term immune mechanism research (the survival time of transplanted cells is limited). The significance is that it improves the defects of traditional SCID mice, it becomes a humanized immune model more close to human DF symptoms, it is suitable for the safety evaluation of dengue fever vaccines and preliminary screening of antiviral drugs, and it provides an in vivo tool for studying antiviral immunity after human hematopoietic stem cell reconstruction.

Hepg2-Transplanted SCID Mice (Spleen Transplanted with Human Hepatocellular Carcinoma Cells): The model was developed by a research team at the Tokyo Metropolitan Institute of Medical Science in Japan [68]. To simulate liver-related pathology of dengue hemorrhagic fever (DHF), human hepatocellular carcinoma cells HepG2 were transplanted into SCID mice through the spleen. After confirming successful transplantation by detecting human serum albumin in the serum, DENV-2 (strain Tr 1751) was intraperitoneally inoculated to establish the model. The advantage is high sensitivity to DENV (the incidence rate of viremia reaches 100% 11 days after infection, with a titer of 10^3.8^–10^5.7^ PFU/mL), which can simulate the core symptoms of DHF (thrombocytopenia, prolonged partial thromboplastin time, increased hematocrit, and elevated serum TNF-α and urea nitrogen); it can also reproduce human hepatocyte-targeted infection characteristics. The limitation is that it only focuses on liver-related pathology, and it cannot simulate the systemic immune response of dengue fever and the complete mechanism of vascular leakage. The immunodeficiency of SCID mice limits the in-depth study of immunopathology of severe dengue fever, and the cell transplantation operation is complex, with the success rate greatly affected by technology. The significance is that it established the first mouse model that can stably simulate DHF symptoms, it provided a key tool for the research on the pathological mechanism of severe dengue fever and the development of targeted antiviral drugs, it filled the gap in models for severe dengue fever (especially those related to liver damage), and it facilitated the breakthrough of severe dengue fever prevention and control technologies.

#### 2.2.3. Gene-Edited Mouse Models

STAT1^−/−^STAT2^−/−^ Double-Knockout Mice: The establishment of STAT1^−/−^STAT2^−/−^ dengue virus (DENV) models originated from the research on JAK-STAT pathway function after the 1990s. Wild-type mice have an intact type I interferon signaling pathway, and mouse STAT2 can resist degradation by the DENV non-structural protein 5 (NS5). STAT2 single-knockout mice still have residual antiviral pathways, making it difficult to reproduce the immune escape process in which interferon signaling is antagonized in human infection, the high viral load-organ damage phenotype of severe dengue fever. Moreover, DENV NS5 only specifically degrades human STAT2 and cannot antagonize mouse STAT2, making it impossible to simulate the interferon signaling inactivation state in human infection. Therefore, it was constructed by crossing and breeding STAT1^−/−^ and STAT2^−/−^ single-knockout mice and screening homozygotes [69,70]. The advantage of this model is that the complete blockage of type I interferon-mediated antiviral signaling through homozygous deletion of key STAT1 and STAT2 genes significantly enhances the infection efficiency of DENV in multiple mouse organs and greatly increases the viral load. It can not only break through species-specific limitations and reproduce the early lethality and multi-organ invasion characteristics of human severe dengue fever but also accurately analyze the division of labor between STAT1-dependent and independent pathways and the core role of interferon signaling in antiviral defense through comparison with single-knockout models. However, the limitation is that the immunodeficiency is too strong, which is different from the physiological state of partial immune activation after human infection, ignoring the synergistic effect of other immune pathways and interferon signaling, resulting in incomplete simulation of infection mechanisms. Its significance is in providing a direct and irreplaceable tool for clarifying the interferon antagonism mechanism of DENV and verifying immune escape targets such as NS5, facilitating the development of antiviral drugs and vaccines targeting interferon signaling pathways or viral replication.

AG129 Mice (IFN α/β/γ R^−/−^): The model was established by van den Broek et al. in 1995 [71]. Based on 129 strain mice, IFN-α/β receptor (IFNAR1) and IFN-γ receptor (IFNGR1) genes were successively deleted by homologous recombination gene knockout technology. In 2006, Shresta et al. first used it for dengue fever research [72]. In 2022, Baldon et al. further verified that it supports the complete “mouse–mosquito–mouse” transmission cycle of various arboviruses such as DENV and ZIKV [73]. The core advantage is the complete lack of IFN-mediated antiviral immunity, which can be infected with multiple arboviruses such as DENV-1~4, ZIKV, and YFV, and it can simulate severe pathologies such as persistent viremia and vascular leakage. The limitation is that the degree of immunodeficiency is much higher than that of humans, lacking a complete immune regulatory network, which is different from the immunopathology of human dengue fever. Its significance is to become the “gold standard” model for the research on dengue fever mosquito vector transmission mechanisms, vaccine efficacy evaluation, in vivo screening of antiviral drugs, and simulation of severe dengue fever pathology, greatly promoting the research and development of dengue fever prevention and control technologies.

hTim4 Transgenic Mouse Model (hTim4-Tg C57BL/6J): The human Tim4 (hTim4) gene and EGFP sequence were inserted into the Tol2 vector and microinjected into fertilized eggs of C57BL/6J mice. Transgenic strains stably passaged only by founders were obtained. They inherited the genetic background of C57BL/6J mice and acquired DENV-2 susceptibility through hTim4 overexpression, retaining complete innate adaptive immunity (the proportions of B, T, and NK cells are consistent with those of wild-type mice) and coagulation function, without pathological distortion related to immunodeficiency [25]. A severe dengue fever (DHF) model can be established through intracranial inoculation of 50 PFU DENV-2 (strain Tr 1751) into 6-week-old mice. The advantage is that the immunocompetent background is close to the human physiological state, and low-dose infection can reproduce core DHF phenotypes (multi-organ hemorrhage, DIC, dengue encephalitis, and intestinal ischemia). The viral load (10^4^–10^7^ copies/mL) is equivalent to that of human severe patients, and it can simulate a Th2-polarized cytokine storm consistent with clinical practice, revealing the Syk-mediated immune coagulation cross-disorder mechanism [73,74]. The limitation is that it is currently mainly suitable for DENV-2, and the susceptibility to other serotypes and variants needs further verification. Intracranial inoculation does not fully simulate the natural transmission mode of mosquito bites, and the regulatory role of mosquito salivary factors is not included. The significance is to break through the bottleneck of traditional immunodeficient models, become the first immunocompetent mouse model that can completely reproduce the DHF pathological chain, provide an accurate platform for the research on dengue fever pathogenesis (especially the severe mechanism of primary infection), be suitable for the verification of vaccine immune response balance and the screening of targeted drugs such as Syk inhibitors, and provide an in vivo tool for the research on severe diseases mediated by multiple mechanisms [75,76].

#### 2.2.4. Humanized Mouse Models

NSG series mice (including NSG, NSG-BLT, NSG-HLA-A2, and NSG-SGM3) are all based on the triple-deficient strain obtained by backcrossing NOD/SCID mice with IL2Rγ^−^/^−^ mice [77]. Their core feature is T/B/NK cell function deficiency. The human immune system is reconstructed by transplanting human CD34+ hematopoietic stem cells (NSG, NSG-HLA-A2, and NSG-SGM3) or combining with human bone marrow/liver/thymus tissue transplantation. Among them, NSG-BLT has more comprehensive immune reconstruction, NSG-HLA-A2 can simulate human HLA-A2-restricted T cell responses, and NSG-SGM3 improves myeloid cell maturity by expressing human SCF/GM-CSF/IL-3. After DENV infection, they can show DF phenotypes such as viremia and thrombocytopenia, suitable for vaccine immunogenicity evaluation, antiviral drug testing, and T/B cell immune mechanism research. Their core advantages are adaptation to DENV clinical isolate infection, immune responses close to humans (such as NSG-BLT, which can produce neutralizing IgM antibodies, and NSG-SGM3, which has more complete human B cell development), diverse application scenarios, and better experimental repeatability. At the same time, there are obvious limitations: none of them can reproduce severe phenotypes such as vascular leakage and multi-organ hemorrhage of DHF/DSS. The engraftment rate of human cells has individual differences of 20–80% with incomplete immune reconstruction. The operation is complex, the experimental cycle is long (4–8 weeks for engraftment), and the feeding cost is high. Except for NSG-SGM3, most models have incomplete humoral immune function (lack of antibody class-switching function). Affected by the source of hematopoietic stem cell donors and engraftment efficiency, there are fluctuations in viral load and cytokine secretion levels among mice of different batches, requiring an increase in sample size to ensure data reliability.

#### 2.2.5. Comprehensive Comparison of Dengue Mouse Models

All dengue virus-susceptible mouse models are developed to overcome mice’s innate antiviral barrier, simulate clinical dengue pathology, and meet differentiated research needs, with modeling principles evolving from utilizing innate immune traits to precise technologies, including gene editing, flora regulation, and humanized immune reconstitution [58,63,72,78]. These models differ in animal strains, infection routes, and pathological simulation focuses, each with unique strengths and limitations, and they have formed a multi-dimensional research system covering age-related susceptibility mechanisms, dengue encephalitis pathology, mosquito-borne transmission regulation, severe dengue pathology, humanized immune response, as well as antiviral drug and vaccine development. It provides hierarchical and targeted in vivo tools for elucidating dengue’s basic mechanisms and developing prevention and control technologies, with highly complementary functional positioning and research scenarios among different models.

Neonatal mouse models rely on the immature immune system of mice aged ≤7 days, serving as exclusive tools for infantile dengue and dengue encephalitis research, filling the gaps in studying age-related susceptibility, DENV neurotropism, and central nervous system injury pathways with safe operation and high reproducibility. Adult immunocompetent mouse adapted strain models and Eh_B17-colonized models free of artificial intervention such as immunosuppression and gene editing act as efficient platforms for large-scale antiviral drug primary screening and host–microbiota–virus interaction research, respectively, with the latter expanding the research direction of dengue transmission intervention [58,60,79]. Immunodeficient mouse models represent the early exploration of humanized models, realizing the upgrade from simple human immune reconstitution to targeted severe pathology simulation; among them, HepG2-engrafted SCID mice are the first to stably simulate DHF liver-related severe pathology, supporting the development of targeted antiviral drugs [63,65,69]. Gene-edited mouse models (STAT1^−^/^−^STAT2^−^/^−^ double-knockout and AG129) break species-specific restrictions by blocking type I interferon-mediated antiviral signaling, effectively simulating severe dengue multi-organ invasion and the complete mosquito–mouse–mosquito transmission cycle, and AG129 mice are hailed as the “gold standard” for dengue model research due to their broad arbovirus adaptability and comprehensive pathological simulation. The hTim4 transgenic mouse model breaks the bottleneck of pathological distortion caused by immunodeficiency in traditional models, being the first to fully replicate the DHF pathological cascade in an immunocompetent background and accurately simulating clinical primary infection-related immune coagulation cross-disorder, providing a human-like research platform for severe dengue mechanism study and targeted drug screening. Humanized NSG series mice with optimized human immune reconstitution are applicable to DENV clinical isolate infection, vaccine immunogenicity evaluation, and human-specific T/B cell immune response research, building a key bridge for the clinical translation of dengue prevention and control technologies [60,69,72,75,78].

Overall, the development of dengue virus-susceptible mouse models shows a core trend from immunodeficiency dependence to immunocompetent adaptation, single pathological simulation to multi-mechanism pathological replication, and basic infection verification to clinical translational application, with continuous improvement in the accuracy, clinical relevance, and application targeting of model construction. However, the existing models still have common urgent optimization problems: most cannot fully replicate core severe phenotypes of DHF/DSS such as vascular leakage and multiple organ hemorrhage [65,73,78,80]; some rely on virus-adapted strains or artificial immune intervention, inconsistent with the physiological background of natural human infection; certain models (e.g., flora-colonized and NSG series mice) suffer from low standardization, large individual experimental differences, and high costs, limiting large-scale application; and some have a single application scenario, such as the intranasal dengue encephalitis model only focusing on central nervous system injury.

### 2.3. Pig Models

Yucatan miniature pigs (*Sus scrofa*) have similar physiological characteristics to humans, lower feeding costs than non-human primates, and abundant immune reagents, making them an important tool for dengue fever research. This model is mainly targeted at DENV-1 serotype, with subcutaneous injection (SC) and intravenous injection (IV) as the main inoculation routes. Subcutaneous inoculation can induce obvious viremia. When re-infected with DENV-1 after subcutaneous inoculation, symptoms similar to human mild dengue fever such as skin rashes and dermal edema will appear. However, intravenous inoculation only produces neutralizing antibodies without significant viremia. DENV immune complexes can be detected in the serum, suggesting the presence of ADE-like phenomena, which is consistent with the immunopathological characteristics of human secondary infection [53,78]. Both inoculation routes can stimulate specific neutralizing antibodies, and the immune response induced by subcutaneous inoculation can also provide protection against subsequent viral challenge. This model fills the gap between small mammal and non-human primate models, but it also has limitations: only the infection data of DENV-1 is clear, the susceptibility to the other three serotypes is unknown, and there are no severe manifestations such as plasma leakage and thrombocytopenia. Overall, the Yucatan miniature pig model provides important support for the research on the pathogenesis of dengue fever and vaccine development. In the future, it is necessary to expand the serotype coverage and optimize the inoculation protocol to improve its application value.

### 2.4. Tree Shrew Models

As small mammals genetically closer to primates, tree shrews (*Tupaia belangeri*) are emerging susceptible models for dengue virus (DENV) infection research, providing important tools for the research on dengue fever pathogenesis and the development of prevention and control measures. This model is mainly targeted at DENV-2 and DENV-3 serotypes, with subcutaneous injection and intravenous injection as the common inoculation routes. After infection, tree shrews will show manifestations similar to human mild dengue fever such as increased body temperature and mild thrombocytopenia. Viral proliferation and brain pathological changes can be detected in the body, and neutralizing antibodies can be produced, which have the basic conditions for vaccine and antiviral drug evaluation [78,81]. Its core advantages are small size, low feeding cost, short reproduction cycle, and high similarity of immune-related genes to humans, making up for the high cost defect of non-human primate models. However, this model also has obvious limitations: the viremia level is extremely low, lacking typical manifestations of human severe dengue fever such as plasma leakage. Wild tree shrews have large individual genetic differences, and tree shrew-specific experimental reagents are scarce, limiting their wide application. In addition, tree shrew lung fibroblasts are susceptible to all four DENV serotypes, and the upregulation of TLR8 gene expression can exert antiviral effects, providing an entry point for in-depth study of host–virus interactions [81]. Overall, the tree shrew model has unique value in basic dengue fever research. In the future, it is necessary to improve the authenticity and stability of simulating human infection by cultivating inbred strains, developing specific reagents, and optimizing virus-adapted strains.

### 2.5. Potential Models

Current evidence of natural infection with dengue virus (DENV) in bats reveals epidemiological characteristics that are regionally positive but geographically heterogeneous. In dengue-endemic areas of Mexico, testing of 19 bat species across five families revealed that four bat species were positive for DENV antibodies, and RT-PCR detected DENV-2, with some individuals simultaneously expressing the NS1 antigen, suggesting acute-phase infection. Furthermore, most positive individuals lacked high levels of neutralizing antibodies, consistent with the phenotype of primary infection [82]. In contrast, nested PCR and haemagglutination inhibition assays conducted on 103 bats in the southeastern and northeastern Brazil failed to detect RNA from arboviruses or flaviviruses (including DENV) or specific antibodies, indicating that bats are not maintenance hosts for DENV in these study areas [83]. Predictions from machine learning models based on phylogeny, environmental factors, and multidimensional geographical distance indicate that various bat species in the tropical regions of the Americas are highly susceptible to DENV, with susceptibility primarily determined by phylogenetic relationships and environmental adaptability. This suggests that more bat species face potential risks of exposure and infection, with high-risk areas concentrated in the tropical belt stretching from central Mexico to northern South America [84]. Taking the existing evidence into account, bats are primarily subject to incidental infection and transient carriage of DENV; most populations do not support sustained viral amplification and circulation and are closer to being epidemiological terminal hosts. Only in dengue-endemic areas characterized by high Aedes mosquito density, the rainy season, and overlapping human outbreaks does a regional risk of cross-species spillover infection exist.

Within the framework of dengue research, bats possess significant potential as model organisms. As natural hosts for a variety of zoonotic viruses, their unique immune tolerance, low inflammatory response, and mechanisms of persistent viral carriage can provide a novel perspective for elucidating DENV’s immune evasion and pathogenic mechanisms. Compared to the high costs of non-human primate models and the age-dependent and viral adaptability limitations of mouse models, primary bat cell lines and in vivo models can more closely mimic natural infection conditions, and they are suitable for assessing viral replication kinetics, tissue tropism, and the early efficacy of candidate vaccines and antiviral drugs [85]. Overall, the characteristics of bats naturally carrying dengue virus make them a key bridge connecting natural transmission and laboratory research, providing a new perspective for the research on dengue pathogenesis and vaccine development. In the future, it is necessary to optimize the model by means of domestication and cultivation, screening of virus-adapted strains, etc., to improve the efficiency of viral replication and the degree of symptom simulation and further tap its application potential as a susceptible model [83].

A variety of animals, such as dogs, horses, birds, marsupials, and bovine animals, have been confirmed to be naturally infected with dengue virus. Their extensive infection evidence and unique biological characteristics give them potential value as dengue fever-susceptible models. As animals closely symbiotic with humans, dogs have a positive rate of 0.65–0.95% detected by RT-PCR in natural surveys in urban and rubber planting areas of Thailand. DENV-2 and DENV-3 serotypes have been successfully isolated, and the virus strains are closely related to local human circulating strains [86]. Their characteristics of convenient feeding and easy sample acquisition provide the possibility for developing models close to human living scenarios. In serological surveys of horses in French Polynesia and New Caledonia, the DENV-1 serum positive rate reaches 6.1–7.7%. Some individuals are simultaneously infected with Zika virus, which can be used to study cross-reactions between dengue fever and other flaviviruses [87]. Dengue-related antibodies have also been detected in horse herds in the Pantanal region of Brazil, further confirming their natural infection potential [88]. Among birds, 11.8% of pigeon samples in the Amazon region of Brazil are HI positive for flaviviruses. The positive rate of dengue antibodies in poultry such as ducks and chickens in Australia, India, and other places is as high as 53.1% [89]. Their advantages of short reproduction cycle and large population size are suitable for batch experimental research. Marsupials (positive rate 13.0%), bovine animals (positive rate 4.1%), and other animals have been detected with dengue nucleic acid or antibodies in systematic reviews in many parts of the world, covering all serotypes from DENV-1 to DENV-4 [84], which can fill the model gap in different ecological niches.

The universality of natural infection, the diversity of viral serotypes, and the advantages of low feeding cost, strong adaptability, and high correlation with humans or the natural environment of these animals provide multiple choices for the development of dengue fever models. In the future, by optimizing detection methods and clarifying viral replication mechanisms, it is expected to develop them into new susceptible models supplementing existing models, facilitating the research on dengue fever prevention and control. A comprehensive overview of commonly used animal models for dengue virus research is summarized in Table 1.

**Table 1:** Animal models for dengue research.

Model Category	Model	Key Characteristics	Limitations	Year	References
Non-human primate (NHP) models	Rhesus monkeys, cynomolgus monkeys, common marmosets, tamarins, bonnet macaques, patas monkeys	Naturally susceptible to DENV, closely mimics human immune responses, exhibits viremia and antibody responses, suitable for evaluating vaccines and therapeutics.	Fails to recapitulate severe human DHF/DSS phenotypes, viral loads are lower than in humans, exhibits inter-species variability and mild clinical manifestations.	1960s (first use)/2000s (standardized)	[44,45,46,47,48]
Immunocompetent mouse models	Suckling mouse model	Permissive to DENV replication, induces transient viremia and lethal encephalitis in neonatal mice with immature immune systems.	Strict age restriction; cannot recapitulate adult DENV pathogenesis, vascular leakage, or DHF/DSS phenotypes.	1990	[58]
	Adult immunocompetent mouse adapted strain model	Mouse-adapted DENV strains achieve efficient replication in adult immunocompetent mice, eliciting robust humoral and inflammatory responses.	Strain/serotype specificity; pathology is primarily neurotropic; fails to mimic human systemic DHF/DSS.	2012	[80]
	Wild-type suckling mouse non-invasive dengue encephalitis model via intranasal infection	Intranasal inoculation of wild-type DENV induces encephalitis and lethality in suckling mice without invasive surgery.	Non-physiological infection route; limited to neonatal mice, not applicable to adult infection studies.	2022	[60]
Immunodeficient mouse models	SCID mouse model	T/B lymphocyte-deficient, supports DENV replication and organ pathology with detectable viremia.	Residual NK cell activity impairs human cell engraftment; cannot model adaptive immunity or ADE-mediated severe disease.	1983	[63]
	NOD/SCID mouse model	Combined NOD background and SCID mutation, with impaired innate immunity, enabling enhanced human hematopoietic stem cell engraftment.	Incomplete human immune reconstitution; lower viral loads and attenuated pathology compared to human infection.	1995	[65]
	HepG2-transplanted SCID mouse model	Human HepG2 cell xenograft supports DENV replication in human hepatocytes, enabling hepatic pathogenesis studies.	Tissue-restricted to liver; cannot recapitulate systemic vascular leakage, hemorrhage, or multi-organ failure of DHF/DSS.	2005	[68]
Genetically engineered mouse models	STAT1^−^/^−^STAT2^−^/^−^ double-knockout mouse model	Ablated type I interferon signaling, highly susceptible to DENV with high viral loads and rapid lethal disease.	Complete loss of innate immunity, pathogenesis deviates from human infection; unsuitable for vaccine or immunotherapy evaluation.	2009	[69]
	AG129 mouse model	Deficient in type I/II interferon responses, permissive to wild-type DENV, recapitulates thrombocytopenia, hemorrhage, and multi-organ injury.	Severe combined immunodeficiency; cannot induce protective immunity or model vaccine efficacy; accelerated disease progression.	1995	[71]
	hTim4 transgenic mouse model	Expresses human DENV receptor hTim4 on an immunocompetent background, supports DENV-2 replication, and fully recapitulates DHF pathological phenotypes.	Restricted to DENV-2 serotype; mechanistic correlation to human ADE and cytokine storm requires further validation.	2025	[74]
Humanized mouse models	NSG series humanized mouse models	NOD/SCID/IL2Rγ^−^/^−^ background with human CD34^+^ HSC engraftment, reconstitutes functional human immune system, and supports DENV replication and human cytokine responses.	Variable engraftment efficiency between individuals; incomplete human immune microenvironment, attenuated DHF/DSS phenotypes compared to clinical infection.	2005–2010	[77]
Pig models	Yucatan miniature pig (*Sus scrofa*)	Physiologically similar to humans; low cost vs. NHPs; abundant immune reagents; mainly targets DENV-1; subcutaneous inoculation induces viremia; secondary infection causes mild dengue-like symptoms and ADE-like phenomena; elicits protective neutralizing antibodies;	Only DENV-1 infection is well-characterized; susceptibility to other serotypes unknown; no severe manifestations such as plasma leakage.	2015	[53]
Tree shrew models	Tree shrew (*Tupaia belangeri*)	Belangeri; small mammal closely related to primates; mainly targets DENV-2/3; infection causes mild dengue-like signs (fever, mild thrombocytopenia); viral replication, pathological changes and neutralizing antibodies detectable; small size, low cost, short reproductive cycle, high similarity in immune genes to humans.	Extremely low viremia; lacks severe dengue hallmarks; large genetic variation; scarce species-specific reagents.	2018	[81]
Bat models	Bat models	Natural reservoir of zoonotic viruses; unique immune tolerance and low inflammation help study DENV immune evasion; primary cell and in vivo models mimic natural infection; suitable for viral kinetics and early drug/vaccine testing.	Mostly incidental and transient infections; do not support sustained viral circulation; require domestication and adapted strain optimization.	2010s (field surveillance)	[82,83,84,85]
	Dogs, horses, birds, marsupials, cattle, etc.	Widespread natural DENV infection covering all four serotypes; low breeding cost, strong adaptability, close linkage to humans or ecological niches.	Require optimized detection methods and clarification of viral replication mechanisms to establish susceptible models.	1970s (first detection)	[84,86,87,88,89]

## 3. Core Bottlenecks of Current Dengue Virus-Susceptible Animal Models

### 3.1. Difficulty in Simulating Core Phenotypes of Severe Dengue Fever

The clinical phenotype spectrum of dengue fever includes dengue fever (DF), dengue hemorrhagic fever (DHF), and dengue shock syndrome (DSS). The core features of severe cases are vascular leakage, bleeding tendency, and shock [32]. Current dengue virus-susceptible animal models generally suffer from insufficient reproduction of key clinical phenotypes. This has become a core bottleneck restricting the development of dengue vaccines, evaluation of antiviral drugs, and in-depth exploration of pathogenic mechanisms.

Vascular leakage stands as the hallmark pathological change in DSS. Clinically, in humans, the diagnostic criteria include a hematocrit (Hct) increase in more than 20%, decreased plasma albumin, and sonographically detectable serous cavity effusion [90]. The simulation ability of the current models remains inadequate. For instance, after AG129 mice are infected with adapted strains, such as D2S10, the Hct can rise by 15% to 20%, approaching the moderate human standard. However, the incidence of serous cavity effusion is less than 10% and lasts for a short period (3 to 5 days, compared with 5 to 7 days in humans). LysM-Cre^+^IFNARfl/fl mice, when in the presence of subneutralizing antibodies, exhibit an Hct increase exceeding 20%, accompanied by obvious plasma leakage and tissue edema, making them one of the best models for simulating vascular leakage to date [91]. Only 5% to 10% of rhesus monkeys show mild serous cavity effusion, with a leakage degree significantly different from that of clinical severe cases [54].

Bleeding/coagulation disorders: Thrombocytopenia can reach diagnostic standards, but active bleeding remains difficult to simulate. The core diagnostic indicators of human DHF are thrombocytopenia (≤100 × 10^9^/L) and coagulation disorders. Among current models, AG129 mice infected with high-virulence strains (such as C0360/94) show a decrease in platelets from 300 × 10^9^/L to 50 to 100 × 10^9^/L, reaching the DHF diagnostic threshold for 3 to 5 days. LysM-Cre^+^IFNARfl/fl mice under ADE conditions have platelets drop below 100 × 10^9^/L, with obvious bleeding manifestations. Only some models, such as AG129 mice infected with DENV-3, show pinpoint or gastrointestinal bleeding. The typical extensive mucosal bleeding seen in humans is absent. Neonatal mice exhibit pinpoint skin hemorrhages (WHO score 1–2) but lack the active bleeding manifestations of human severe cases, such as gingival bleeding and gastrointestinal bleeding [58,91].

The cytokine storm in human severe dengue fever is characterized by TNF-α levels of 50 to 100 pg/mL, IL-6 levels of 80 to 150 pg/mL, and peaks 3 to 5 days after onset. In current models, serum IL-6, IFN-γ, and TNF-α levels in AG129 mice can increase five to 10 times after infection, with a significant elevation in IL-10, but the peak times vary inconsistently—some models show peaks at 5 to 7 days, slightly delayed compared with humans. Humanized mice (such as NSG and its derivative models) can produce human-specific cytokines (IFN-γ, IL-2, and vascular endothelial growth factor), but IL-6 and TNF-α levels are usually lower than those in severe patients [91].

### 3.2. Insufficient Restoration of Immune Mechanisms: Failure to Simulate the Immunopathological Process of Natural Infection

The development of dengue virus-susceptible animal models has progressed from passive adaptation relying on single phenotype simulation and immune deficiency to active breakthroughs toward comprehensive and accurate reproduction. Initially, only single phenotype simulation was achievable. For example, even with high-dose DENV inoculation, wild-type C57BL/6J mice rarely exhibit the core severe phenotypes such as vascular leakage and coagulation disorders [80], and non-human primates only develop viremia without obvious clinical symptoms. A breakthrough was later made to simulate partial severe phenotypes. AG129 mice, with knocked-out IFN-α/β/γ receptors, can systematically simulate the core pathological features of DHF/DSS, but their fundamental limitation lies in the lack of natural immune temporal activation and adaptive immune memory formation, making them unsuitable for vaccine immunogenicity evaluation and ADE mechanism research [71,72,73].

The latest generation of models, represented by hTim4 transgenic C57BL/6J mice, retain intact innate immune pathways with normal IFN-α/β/γ signaling. They can normally activate T/B/NK cell responses and produce specific neutralizing antibodies, clarifying that primary infection can induce severe cases through Syk-mediated immune coagulation cross-disorder and revising the traditional understanding that ADE is the sole driver of severe disease [25]. Notably, their susceptibility to low doses (50 PFU) is close to human natural infection doses, and phenotypes such as multi-organ hemorrhage and encephalitis can be directly used for vaccine protective effect evaluation and antiviral drug screening. The Syk-Th2 pathway they revealed provides a clear target for targeted therapy, such as Syk inhibitor repurposing. However, problems still exist, including the lack of unified standards for inoculation methods, inoculation doses, and evaluation indicators (such as cytokine detection time points and vascular leakage quantification standards), as well as fluctuations in cytokine storm intensity among different experimental batches.

The applicability of current DENV-susceptible animal models highly depends on multiple variables, including host species, immune status, viral serotype/variant, vaccine dose, inoculation route, and infection frequency. These variables lead to significant differences and poor comparability among models. The core bottleneck of current research is solving the in vivo simulation of ADE and ensuring consistent simulation of cytokine storms. ADE is a core mechanism of severe dengue fever, particularly associated with sequential heterotypic DENV infection and the infection in seronegative individuals after vaccination [92].

When humans experience sequential heterotypic DENV infection, specific antibodies induced by primary infection decline to subneutralizing levels and form immune complexes with heterologous viruses. These complexes mediate massive viral replication in macrophages and monocytes by binding to FcγR receptors on immune cell surfaces, while activating excessive inflammatory responses [93,94]. The infection risk in seronegative individuals after vaccination further highlights its importance. As the first approved tetravalent vaccine, Dengvaxia failed to fully predict the ADE risk in seronegative recipients, leading to a significant increase in hospitalization rates after natural infection and ultimately prompting the WHO to restrict its use to seropositive individuals.

Consistent simulation of cytokine storms is a key link connecting ADE mechanisms and severe phenotypes. The clinical data confirm that ADE-mediated severe dengue fever is often accompanied by intense Th2-polarized cytokine storms. The explosive increase in core factors such as IL-6, IL-10, and TNF-α directly exacerbates vascular endothelial damage, vascular leakage, and multi-organ dysfunction. Additionally, in severe human DENV infection, IL-10 levels increase, while interferon levels decrease [95]. In contrast, current animal models show elevated levels of both IL-10 and interferons. This inconsistency in cytokine storm simulation makes it impossible to accurately verify whether candidate vaccines can simultaneously induce protective neutralizing antibodies and balanced immune responses and to precisely identify targeted intervention drugs for core regulatory nodes of the storm.

It also hinders the determination of the optimal drug intervention window, severely restricting the development of targeted therapies for severe dengue fever [91]. Species differences in Fc–Fc receptor interactions further exacerbate simulation difficulties. The key molecular basis of human ADE is FcγRIIa (CD32a)-mediated internalization of virus–antibody complexes [96]. However, mouse Fcγ receptors have low affinity for human IgG, different signaling pathways, and distinct expression profiles. Even in humanized mice (NSG-hu) [77], the implanted human monocytes/macrophages express human FcγRIIa, but their cell proportion, activation status, and tissue distribution differ from those in humans, leading to unpredictable ADE sensitivity and enhancement. The lack of human FcγR knock-in models prevents accurate simulation of the molecular details of antibody–Fc receptor interactions.

### 3.3. Unclear Viral Receptor Mechanisms Restrict Model Optimization Directions

The receptor mechanism of DENV cell entry is the theoretical basis for constructing genetically modified susceptible models. However, the field currently faces the dilemma of “numerous candidates, unclear functions, and insufficient in vivo evidence,” resulting in ambiguous model construction directions and further exacerbating the chaos in the model system [97]. Potential receptors (such as AXL, CLEC5A, FcγR, and DC-SIGN) are mostly supported by in vitro evidence with insufficient in vivo functional verification [20].

As a member of the TAM receptor family, AXL mediates DENV-2/3 entry into various cell lines in vitro, but no mouse model with AXL expression has been used to verify its in vivo necessity. As a pattern recognition receptor, CLEC5A can interact with DENV in vitro, and only partial in vivo evidence in STAT1-deficient mice supports its involvement in inflammatory responses, but it is not essential for infection. DC-SIGN/L-SIGN are classic DENV-binding molecules on dendritic cells, but no mouse model expressing human DC-SIGN has been used to verify in vivo infection dependence [67,98]. The role of FcγR in ADE is widely accepted, but direct evidence from genetically modified models is lacking to confirm whether specific FcγRIIa blocking can inhibit ADE in vivo [99].

These receptors have clear biological activities in vitro, but their actual in vivo effects remain controversial, leading to ambiguous research directions for genetically modified models constructed based on these receptors. Although receptor overexpression can enhance host susceptibility to pathogens, it is difficult to fully replicate the pathophysiological process of the disease. While receptor knockout can alleviate the host inflammatory response, it may interfere with the natural and immune clearance of viruses by the host.

Receptor functional redundancy and tissue specificity increase the complexity of the mechanism analysis. DENV may use multiple receptors to enter different cell types: hepatocytes may mainly rely on CLEC5A and AXL, monocytes on DC-SIGN and FcγR (under ADE conditions), and endothelial cells on CLEC5A and integrin β3. Overexpression or knockout of a single receptor may not be sufficient to alter overall susceptibility, requiring combined modification of multiple receptors, but the synergistic or compensatory relationships between receptors remain unknown [100,101,102].

For example, after AXL knockout, Mer or Tyro3 may be upregulated compensatorily; after CLEC5A knockout, TLR2/4 signaling may be enhanced. This redundancy makes it difficult to establish a simple correspondence between receptors and phenotypes, complicating target selection for model construction. The amino acid difference in the envelope (E) protein among the four DENV serotypes (DENV-1~4) reaches 25% to 40%, with significant differences in receptor-binding sites, making it difficult for current models to achieve broad-spectrum susceptibility [103,104,105].

The current models are unable to replicate the complete pathological chain of DHF/DSS clinical features, including multi-dimensional manifestations such as gingival bleeding, hepatomegaly, progressive vascular leakage, and shock. On the one hand, the inoculation route deviates from natural transmission. The hTim4 model uses intracranial inoculation, which can induce multi-organ hemorrhage and disseminated intravascular coagulation (DIC) but fails to simulate the immune surveillance process of the body against peripherally invading pathogens during the mosquito-borne subcutaneous infection. The impact of mosquito salivary factors (such as adenosine deaminase) on immune regulation is overlooked, leading to deviations in the pathological initiation mechanism from human natural infection.

On the other hand, a simulation of key clinical indicators is insufficient. Clinical DSS vascular leakage progresses progressively, with an early perfusion pressure decrease of 10% to 20% and a late decrease exceeding 30%. However, most current models focus on terminal symptoms and lack stable simulation of early warning signal stages, such as progressive thrombocytopenia and mild ALT/AST elevation, making them unable to meet the screening needs of preclinical early intervention drugs [25].

### 3.4. Insufficient Model Standardization and Practicality: Lack of Unified Inoculation Doses and Evaluation Indicators

The core manifestations of insufficient standardization and practicality of DENV mouse models lie in two aspects. In terms of inoculation doses, the dose range spans six orders of magnitude (10^2^–10^8^ PFU) among models with different genetic backgrounds, such as wild-type, AG129, and humanized mice, as well as among different studies using the same model. There is no recognized benchmark for selecting inoculation routes (intracranial, intraperitoneal, subcutaneous, etc.) and doses, leading to differences in dose–effect relationships.

In terms of evaluation indicators, there are no unified standards for the detection dimensions, time points, and judgment thresholds of clinical phenotype assessment (survival rate, and physical sign grading), pathophysiological indicators (viral load, vascular leakage degree, liver and kidney function markers, and blood cell count), and immunological markers (cytokine profile, neutralizing antibody titer, and PRNT_50_), resulting in the absence of a standardized evaluation system [52,73,77].

## 4. Prospects of Dengue Virus-Susceptible Animal Models

The future development of DENV-susceptible animal models can leverage the fact that most existing models only simulate partial or single severe DENV phenotypes, fully exploring their value through precise experimental design. First, hierarchical animal models should be constructed according to research purposes, using targeted models to verify the effectiveness of antibodies or vaccines. These models can then be used to explore the pathogenesis of severe cases in depth.

Subsequently, humanized replacement or pathway simulation can verify the uniqueness of pathogenic mechanisms, gradually piecing together and improving the complete pathogenesis of severe dengue fever in a step-by-step manner to maximize the practical value of existing models. At the same time, the focus will be on simulating ADE and cytokine storms, while meeting four core requirements: intact immunity, broad-spectrum susceptibility, accurate simulation of ADE and cytokine storms, and consistency with clinical phenotypes.

By addressing current bottlenecks, a model capable of restoring core human DHF/DSS phenotypes, such as systemic hemorrhage, progressive vascular leakage, and organ failure, should be constructed. This model should also resolve the poor comparability of inoculation doses (closer to human natural infection levels), clinical phenotypes, and pathological indicators among different models. It will provide a scientific and practical experimental tool for the development of tetravalent DENV vaccines, screening of targeted drugs, and research on the pathogenesis of severe cases, accelerating the global progress of dengue fever prevention and control.

## 5. Conclusions

Dengue is a major mosquito-borne viral disease of global public health importance, with its epidemic spreading globally and increasing transmission risk in temperate regions. Current prevention and control measures are constrained by limited vaccine applicability, lack of specific antiviral drugs, and mosquito vector resistance, making effective control challenging. Dengue virus-susceptible animal models are critical for elucidating pathogenesis, developing vaccines, and advancing control strategies. Non-human primate models, with immune responses similar to humans, serve as core tools for preclinical vaccine evaluation. Mouse models, the most mature system, include various subtypes that facilitate mechanistic research and preclinical testing, while emerging models (e.g., swine, tree shrews) fill gaps in ecological and experimental needs but require further optimization for broader applicability. Key challenges remain in model development: difficulty in fully replicating severe disease phenotypes, insufficient functional validation of viral entry receptors, and lack of standardized protocols, which limit experimental reproducibility. Future efforts should focus on developing immunocompetent, serotype-broad models that accurately mimic human pathological features, establishing unified evaluation standards and enhancing the scientific rigor and practicality of models to support vaccine development, therapeutic screening, and mechanistic research, ultimately advancing global dengue control.

## Data Availability

Not applicable. All data presented in this review are sourced from the cited published literature, and no new original data were generated in this work.
